# Exploring the unfolding pathways of protein families using Elastic Network Model

**DOI:** 10.1038/s41598-024-75436-8

**Published:** 2024-10-13

**Authors:** Ranjan Kumar, Sandipan Dutta

**Affiliations:** Department of Physics, Birla Institute of Technology and Science, Pilani, Rajasthan 333031 India

**Keywords:** Biophysics, Computational biophysics, Biological physics

## Abstract

We explore how a protein’s native structure determines its unfolding process. We examine how the local structural features, like shear, and the global structural properties, like the number of soft modes, change during unfolding. Simulations are performed using a Gaussian Network Model (GNM) with bond breaking for both thermal and force-induced unfolding scenarios. We find that unfolding starts in areas of high shear in the native structure and progressively spreads to the low shear regions. Interestingly, analysis of single domain protein families (Chymotrypsin inhibitor and Barnase) reveal that proteins with distinct unfolding pathways exhibit divergent behavior of the number of soft modes during unfolding. This suggests that the number of soft modes might be a valuable tool for understanding thermal unfolding pathways. Additionally, we found a strong link between a protein’s overall structural similarity (TM-score) and its unfolding pathways, highlighting the importance of the native structure in determining how a protein unfolds.

## Introduction

The dynamic process of protein folding, in which a linear polypeptide chain transforms into a functional three-dimensional structure is an important phenomenon in molecular biology. The unfolding of the protein, on the other hand, typically involves the loss of the structure through the disruption of various stabilizing interactions, such as hydrogen bonds, hydrophobic interactions, and disulfide bonds^1–7^. This is a multi-step process involving formation of many intermediate states before it completely unfolds into the linear chain. It has been observed for small monomeric proteins that the folding and unfolding pathways share striking similarities, hence studying protein unfolding can provide insights into the folding pathways^8,9^. Moreover, the study of unfolding pathways can also lead to a better understanding of the progression of diseases associated with protein misfolding^10^.

In recent years, considerable progress has been made through theoretical and experimental approaches that provide insights into the factors leading to denaturation. Spectroscopic technique such as circular dichroism has been used to study the unfolding and folding of proteins as a function of temperature^11^. Fluorescence Spectroscopy^12^, single-molecule techniques^3,13–16^, small angle X-ray scattering^17^ and Nuclear Magnetic Resonance^18^, have contributed valuable insights into protein folding/unfolding dynamics. Theoretical investigations have predominantly relied on Molecular Dynamics (MD) and Monte Carlo (MC) simulations to study unfolding phenomena^19,20^. Probing the intricate nature of protein folding and unfolding both experimental and theoretical investigations have consistently revealed the wealth of information concealed within the native-state topology. The native structure plays a pivotal role in determining the speed and mechanisms of protein folding^21–23^. Proteins with similar folds but distinct sequences often exhibit comparable folding rates^23^. To simulate protein unfolding, researchers have devised a method that involves systematically breaking hydrogen bonds and salt bridges within the native structure, taking into consideration their relative energies. In the pursuit of unraveling protein unfolding, a graph-based approach has proven valuable for identifying rigid and flexible regions within proteins, shedding light on the dynamic changes in flexibility during the process^24,25^. This collective body of work strongly underscores the critical role of the native-state topology in governing protein folding and unfolding mechanisms.

In this work we delve into the structural changes proteins undergo during unfolding, focusing on both local and global transitions. While some previous research have explored local rigidity changes during unfolding^26^, a complete understanding of the interplay between the local and the global structural changes remains elusive. We introduce the shear part of the strain^27–29^, a readily calculable parameter, to identify “floppy” regions within the protein structure. These floppy regions are more prone to unfolding and could be potential drug targets in diseases associated with protein misfolding. Proteins belongs to a larger classes of materials called amorphous materials and the soft vibrational modes can be used to identify the soft regions in amorphous materials that are prone to rearrangement during shear deformation^30–34^. They also play a crucial role during glass transition^35–38^ and the shear localization which can lead to global structural failure in the system^39–42^. Rader et. al.^24^ showed that the global structural property, the number of floppy modes, can capture the rigidity transition in proteins during unfolding. They found a sharp first-order-like transition from rigid to floppy regime. Here we show that the number of floppy modes contains much more information than just the rigidity to floppy transition. Our unfolding simulations of the members of protein families show that the unfolding is a gradual process occurring through intermediate unfolding pathways and that the number of soft modes can effectively differentiate the unfolding pathways for various proteins. Our simulations also establish a strong connection between the native structure of a protein and its unfolding pathways.

In our work we simulate unfolding through a bond breaking algorithm based on GNM developed by Su et. al.^43^. GNM belong to a larger class of models called Elastic Network Models (ENM)^43–57^ that are useful in the study of the elasticity properties of proteins. These models along with Normal Mode Analysis successfully capture the large scale functional dynamics of proteins about their native structure. The residues are represented by their $C_{\alpha }$ atoms, while the detailed interactions between residues in their native states are approximated using springs. In the GNM based algorithm, the bonds experiencing the highest fluctuations under the influence of thermal or external forces are broken leading to a gradual unfolding of the protein^51,58^. Remarkably, the simplified model based on GNM accurately capture the bond breaking sequence of atomistic MD simulations^43,59,60^. Our focus would be on the families of monomeric proteins CI2 and Barnase for which the GNM method has been well tested^43^.

## Methods

### Unfolding simulations

We perform unfolding simulations of proteins based on the GNM model of Su et. al.^43^. GNM is a coarse grained model where the protein is represented as a bead-spring network with residues as beads. The $\alpha$-Carbon atoms of the residues within a certain cutoff distance $r_c = 7$ Åare connected by springs of the same spring constant $\kappa$. The covalently bonded pairs along the chain backbone have a spring constant $c\kappa$. The parameters *c* and $\kappa$ for the protein are obtained by fitting the B-factors with the experiments. Given that the springs are harmonic and the residue fluctuations are isotropic and Gaussian, the overall conformational potential of the structure can be written as

1 $$\begin{aligned} {V}=\frac{\kappa }{2}\sum _{i,j}^N\left[ \Delta \textrm{x}_i\Gamma _{ij}\Delta \textrm{x}_j +\Delta \textrm{y}_i\Gamma _{ij}\Delta \textrm{y}_j+\Delta \textrm{z}_i\Gamma _{ij}\Delta \textrm{z}_j\right] , \end{aligned}$$

where $\Delta {x}_i = x_i-x_i^{0}$ is the fluctuations of the *x*-coordinate of the *i*th residue about its equilibrium position $x_i^{0}$. *N* is the number of residues in the protein; $\Gamma _{i j}$ are the elements of the Kirchhoff matrix also called connectivity in graph theory and in our case takes the values

2 $$\begin{aligned} \Gamma _{ij} = {\left\{ \begin{array}{ll} -c& \hspace{5mm}\text {if }\vert i-j\vert =1 \\ -1& \hspace{5mm}\text {if }\vert i-j\vert>1\text { and }r_{ij}<r_c \\ 0& \hspace{5mm}\text {if }\vert i-j\vert>1\text { and }r_{ij}>r_c \\ -\sum _{i,j\ne i}\Gamma _{ij}& \hspace{5mm}\text {if }i=j. \\ \end{array}\right. } \end{aligned}$$

$$r_{i j}$$ is separation between the $$C_{\alpha }$$ atoms of the $$i^{\textrm{th}}$$ and $$j^{\textrm{th}}$$ residues and $${r_c}$$ is cutoff distance. We use the same values of $$c = 9.3$$ and $$\kappa = 0.493$$$$k_BT/$$Å^2^ for all the proteins of the 2CI2 family, and $$c = 2.3$$ and $$\kappa = 0.945$$$$k_BT/$$Å^2^ for all proteins of the Barnase family in our work^43^.

The probability distribution of fluctuations $\Delta {\textbf{R}}_i=[\Delta {x}_i, \Delta {y}_i,\Delta {z}_i]$ about the native structure are Gaussian $p(\{{\textbf{R}}_i\}) \propto \exp (-\frac{\kappa }{2k_BT}V)$ and isotropic along *x*, *y* and *z* directions. $k_B$ and *T* are the Boltzmann constant and the temperature of the water environment. Using this, the cross-correlations fluctuation between $i^{\textrm{th}}$ and $j^{\textrm{th}}$ residue can then be shown to be

3 $$\begin{aligned} \langle \Delta {\textbf{R}}_{i}\cdot \Delta {\textbf{R}}_{j}\rangle =\frac{3k_{\textrm{B}}T}{\kappa }[\Gamma ^{-1}]_{i j}, \end{aligned}$$

where $\Gamma ^{-1}$ is the inverse of $\Gamma$. From this we obtain the mean-square fluctuation in the distance vector $R_{ij}$ between the residues $i^{\textrm{th}}$ and $j^{\textrm{th}}$ to be

4 $$\begin{aligned} \langle (\Delta R_{\textrm{ij}})^{2}\rangle =\left\langle \left( {\textbf{R}}_{\textrm{ij}} -{\textbf{R}}_{\textrm{ij}}^{0}\right) ^{2}\right\rangle ={\frac{3k_{\textrm{B}}T}{\kappa }}\left( [\Gamma ^{-1}]_{\textrm{ii}} +[\Gamma ^{-1}]_{\textrm{jj}}-2[\Gamma ^{-1}]_{\textrm{ij}}\right) . \end{aligned}$$

${\textbf{R}}_{\textrm{ij}}$ and $\textstyle {\textbf{R}}_{ij}^{0}$ are the instantaneous and equilibrium separation vectors between $i^{\textrm{th}}$ and $j^{\textrm{th}}$ residues.

For thermal unfolding, the temperature is increased and the bonds in the protein which undergo large fluctuations start breaking. All the forces are then redistributed among the other surviving bonds altering the fluctuations of the remaining bonds. Thus the unfolding of proteins is a highly nonlinear process. The following algorithm uses GNM for thermal unfolding of proteins

1. The mean square fluctuations between all bonds connecting the $\hbox {C}_{\alpha }$ atoms from matrix $\Gamma$ in Eq (4).
2. The bond with the largest fluctuation between residues *i* and *j* is broken, and matrix $\Gamma$, which represents a new topology during protein unfolding, is recalculated: $\Gamma _{ij}\rightarrow \Gamma _{ij}+1$, $\Gamma _{ii}\rightarrow \Gamma _{ii}-1$ and $\Gamma _{jj}\rightarrow \Gamma _{jj}-1$.
3. The mean square fluctuations of the distance between all residue pairs are calculated using the new matrix $\Gamma$.
4. The above steps are repeated until all non-backbone bonds are broken.

To study force-induced unfolding, we consider a protein being acted on by an external force $\textbf{f}$. Within the GNM we can obtain the *x* component of the displacements $\textbf{u}$ of the residues from the Hooke law

5 $$\begin{aligned} {\textbf {u}}_x=\kappa ^{-1}\Gamma ^{-1} {\textbf {F}}_x. \end{aligned}$$

The *i*th element of the vectors ${\textbf{u}}_x$ and ${\textbf{F}}_x$ store the *x* component of the displacement and the force on *i*th residue respectively. Similar expressions for the Hooke law hold for the *y* and *z* components. Here we apply unit force along the *x* direction at the end residues, ${\textbf{F}}_x(1) = -1$ and ${\textbf{F}}_x(N) = 1$.

We use the following algorithm for force-induced bond breaking

1. First the displacements of the residues are calculated from Eq (5) for a given applied force.
2. The bond with the largest stretching $\Delta u_{i j}=\left| {\textbf {u}}_{i}-{\textbf {u}}_{j}\right|$ is broken and the connectivity matrix $\Gamma$ is updated: $\Gamma _{ij}\rightarrow \Gamma _{ij}+1$, $\Gamma _{ii}\rightarrow \Gamma _{ii}-1$ and $\Gamma _{jj}\rightarrow \Gamma _{jj}-1$.
3. The above steps are repeated until all the non-backbone bonds are broken.

In this algorithm the network progressively becomes more flexible and the bond breaking is accelerated as they get stretched more under the fixed external force.

For all members of CI2 (Barnase) family, we stop both the thermal and the force-induced simulations after 110 (200) bonds are broken.

### Soft modes

Anisotropic Network Model (ANM) is another kind of ENM^57,61^ based on spring network similar to GNM. The potential of this network is $\frac{\kappa }{2}\sum _{j<i}\Gamma _{ij}\left( R_{ij}-R_{ij}^0\right) ^2$. The Hessian *H* of this network is the second derivative of this potential with respect to the position of the residues. The Hessian matrix *H* is defined in terms of $3\times 3$ blocks

6 $$\begin{aligned} H_{ij} = \kappa \Gamma _{ij}{\textbf{n}}_{ij}{\textbf{n}}_{ij}^T, \end{aligned}$$

for $i\ne j$, where ${\textbf{n}}_{ij} = {\textbf{R}}^0_{ij}/R^0_{ij}$ is the unit vector along the bond joining the $\hbox {C}_\alpha$ atoms of *i*th and *j*th residues. The diagonal blocks of *H* are calculated as $H_{ii} = -\sum _{j\ne i}H_{ij}$.

The eigenvalues $\lambda _i$ of the Hessian matrix *H* of ANM are positive or zero. The eigenvectors corresponding to the zero eigenvalues are called the floppy modes. In this work, we consider an eigenmode floppy if its eigenvalue is less than $10^{-4}$. The translation and rotational symmetries of the matrix *H* always result in six zero eigenvalues, while the presence of more floppy modes signifies that the protein is under-constrained.

### Calculation of shear

Consider a protein with the coordinates of residues in the crystal structure ${\textbf{x}}$ and of the deformed state ${\textbf{x}}^{\prime }=\chi ({\textbf{x}})$. The local deformation of the coordinates ${\textbf{x}}$ is given by ${\textbf{F}} = \frac{\partial {\textbf{x}}^{\prime }}{\partial {\textbf{x}}}$. In the discrete form, the deformation matrix depends on the un-deformed relative positions of the *m*th residue and its neighboring *n*th residues $\Delta {{\textbf{x}}}^{mn}={\textbf{x}}^n-{\textbf{x}}^m$, the deformed positions $\Delta {{\textbf{x}}^{\prime }}^{mn}={{\textbf{x}}^{\prime }}^n-{{\textbf{x}}^{\prime }}^m$ and a weight function $w_n$. The results have no strong dependence on the weight functions provided the neighborhoods are chosen large enough. Here we choose a radial weight function

7 $$\begin{aligned} w_{mn} = {\left\{ \begin{array}{ll} 1 & r_{mn} \le 6~\text{\AA }\\ 1-\frac{1}{2}(r_{mn}-6) & 6~\text{\AA }< r_{mn} \le 8~\text{\AA }\\ 0 & r_{mn} \ge 8~\text{\AA }, \end{array}\right. } \end{aligned}$$

where $r_{mn} = \vert \Delta {\textbf{x}}^{mn}\vert$. It has the expression ${\textbf{F}}_m = {\textbf{A}}_m{\textbf{D}}_m^{-1}$, where ${\textbf{A}}_m = \sum _n\Delta {{\textbf{x}}^{\prime }}^{mn}\Delta {{\textbf{x}}^{mn}}^Tw_{mn}$ and ${\textbf{D}}_m = \sum _n\Delta {\textbf{x}}^{mn}\Delta {{\textbf{x}}^{mn}}^Tw_{mn}$. The Eulerian strain tensor is calculated as $\epsilon _m = \frac{1}{2}\left[ {\textbf{I}}-({\textbf{F}}_m{\textbf{F}}_m^T)^{-1}\right]$^27,62^, where ${\textbf{I}}$ is the identity $3\times 3$ matrix. The strain tensor is then $\gamma _m = \epsilon _m-\frac{1}{3}(Tr\epsilon _m){\textbf{I}}$. The shear component of the elastic energy of deformation at the residue *m* is $s_m = \sum _{i,j}^3(\gamma _m)_{ij}^2$. In our work we calculate the total shear $s_m$ at residue *m* by summing over the contribution of the shear due to each soft modes i.e. choosing ${\textbf{u}}$ as a soft eigenmode calculated in Sec 2.2. If a fraction *f* of the 3*N* modes are softmodes, the total shear is obtained by summing over these 3*fN* softmodes. As the protein becomes more floppy during unfolding, the fraction *f* becomes larger.

### Correlation matrices

To determine whether two proteins unfold along similar pathways, we compare the sequence in which the bonds between their secondary structures break. For CI2 with an $\alpha$-helix, three $\beta$-strands and two loops, we obtain $N_r = 5$ regions by cluster analysis of the contact map for the CI2 protein family corresponding to the interactings between the different secondary structures. To obtain a quantitative description of the bond breaking sequence, we define $N_r$ dimensional vectors $v_r$ which has 1 at one position and zero everywhere else. For example, for CI2 we use $v_1=[1,0,0,0,0]$ for N-terminus- $\beta _3$, $v_2=[0,1,0,0,0]$ for $\alpha$-$\hbox {Loop}_2$, $v_3=[0,0,1,0,0]$ for $\beta _1$-$\beta _2$, $v_4=[0,0,0,1,0]$ for $\beta _2$-$\beta _3$ and $v_5=[0,0,0,0,1]$ for the rest of the non-covalent bonds. The individual bond breaking events are categorized into region-wise vector representation. For each protein, the $N_b$ bond breaking sequence is transformed into $N_b\times N_r$ which is flattened to a $N_bN_r\times 1$ vector. For $N_p$ proteins in the family we obtain a matrix $N_p\times N_bN_r$ from which the covariance matrix is calculated. For the Barnase family we get $N_r = 30$ regions in the contact map based on the interactions between the N-terminus, the three $\alpha$-helices, the three $\beta$-strands and the two loops.

## Results and discussion

### Shear evolution and floppy modes analysis - local and global parameters

The weakest regions of a protein, that are more susceptible to unfolding, can be obtained from the calculation of strain^27^, which measures the local deformation in an elastic medium from the spatial gradient of displacement^62^. If the coordinates of an elastic medium is deformed by ${\textbf{x}}\rightarrow {\textbf{x}}^{\prime }={\textbf{x}}+{\textbf{u}}$, strain is calculated as

8 $$\begin{aligned} \varepsilon _{i j}={\frac{1}{2}}\left( {\frac{\partial u_{i}}{\partial x_{j}}}+{\frac{\partial u_{j}}{\partial x_{i}}}~-{\sum _{k}}{\frac{\partial u_{k}\partial u_{k}}{\partial x_{i}\partial x_{j}}}\right) , \end{aligned}$$

where $u_{i}$ is the $i^{\textrm{th}}$ component of the displacement vector ${\textbf{u}}$. The strain tensor is the second rank tensor ( similar to $3\times 3$ matrix ) which has six distinct components that describe entirely the local stretching or compression, twisting, and shear phenomena occurring at a specific location within the structure. The shear tensor is obtained from the strain tensor using

9 $$\begin{aligned} \gamma =\varepsilon -\textstyle {\frac{1}{3}}(\textrm{Tr} {\epsilon )}\ {\mathbb {I}}, \end{aligned}$$

where ${\mathbb {I}}$ is the $3\times 3$ identity matrix. The shear energy is the sum of all elements of the shear matrix $\sum _{i,j}\gamma _{ij}$ (Sec 2.3). The displacements from the floppy modes are calculated in Sec 2.2 and are used to obtain the total shear energy (Sec 2.3). From now on we call the total shear energy as the shear. The most flexible regions of the protein are regions with high shear.

**Fig. 1:**
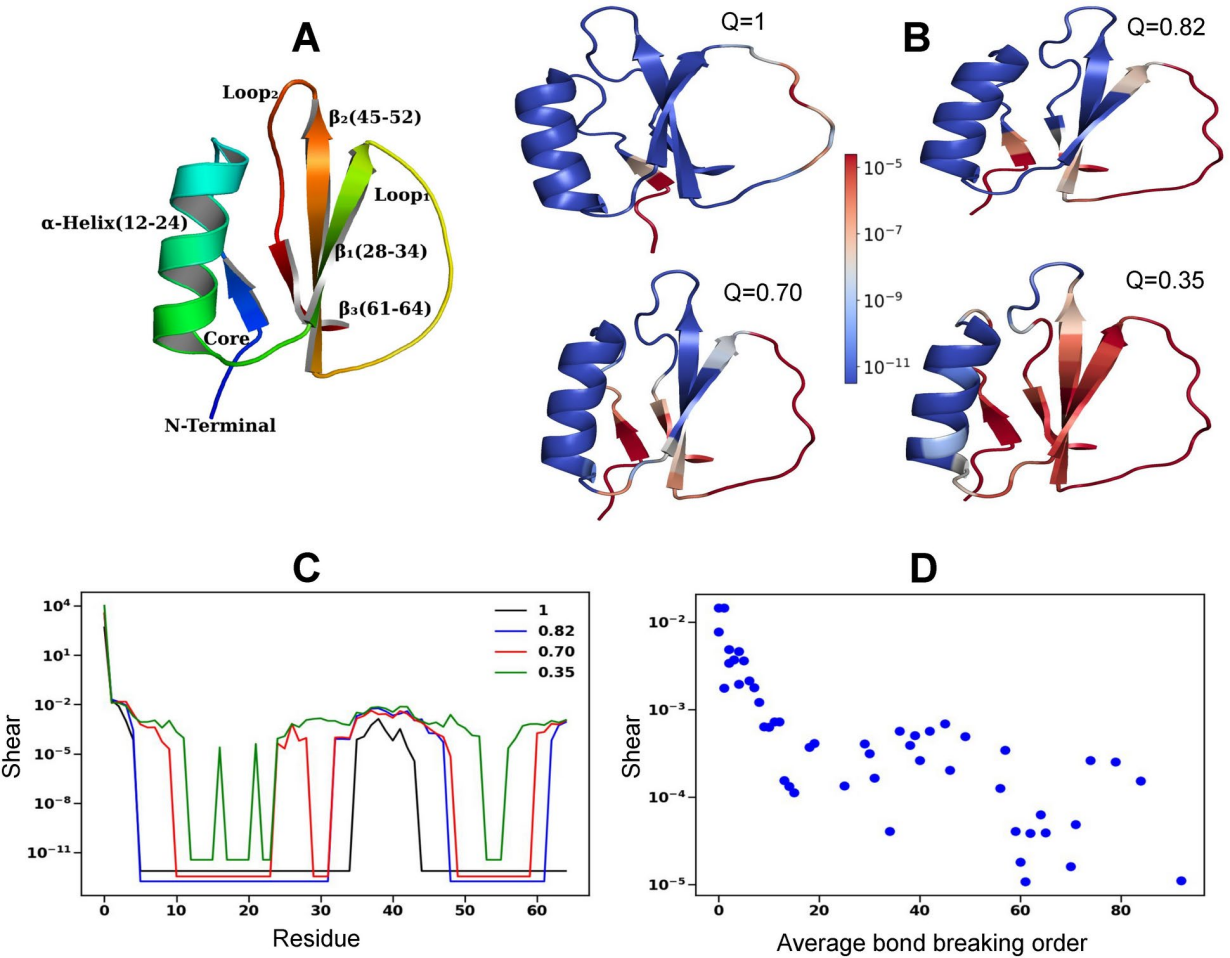
Thermal unfolding of CI2. (**A**) The secondary structures in the native state of CI2 (PDB ID: 2ci2). (**B**) Shear for different *Q* values, which is the fraction of native contacts, at the different stages of unfolding. Regions of high (low) shear are shown in red (blue). (**C**) The shear at each residue at different *Q* values. (**D**) The scatter-plot of the shear at the residues in the native structure (*Q* = 1 in (**B**)) vs corresponding the average order of bonds breaking. The average bond-breaking order for each residue was calculated by averaging the order at which its individual bonds to neighboring residues are broken during a simulation of 110 bond ruptures within the protein.

**Fig. 2:**
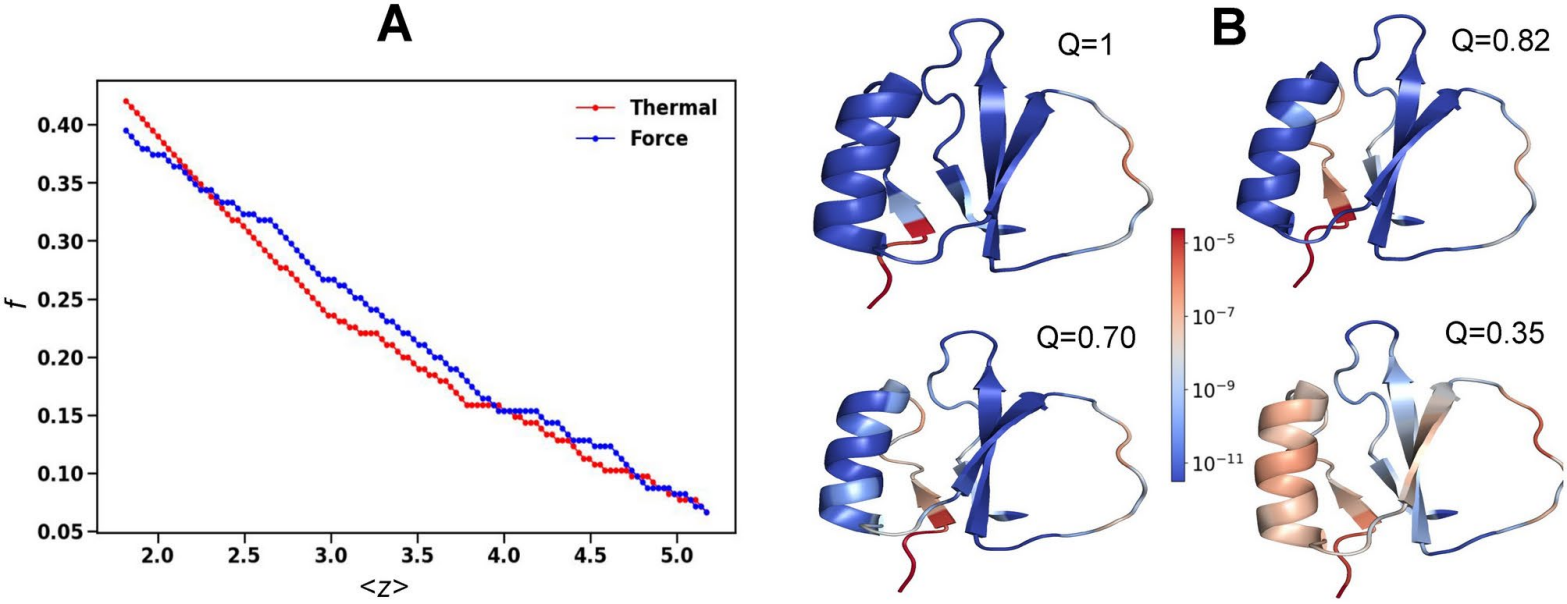
Force-induced unfolding of CI2. (**A**) The number of floppy modes, *f*, as a function of the average coordination number of the residues, $\langle z\rangle$ for thermal and force unfolding of CI2. (**B**) The shear at each residue during unfolding for different *Q* values. Regions of high (low) shear are shown in red (blue).

The native structure of the extensively studied Chymotrypsin inhibitor-2 (CI2) (PDB code 2ci2), consists of an $\alpha$-helix and three-strand $\beta$-sheet as shown in Fig 1-A. In the native structure, the shear peaks at the hinge site ( at residue 38 in the black curve in Fig 1-C in the native state) as well as at the N-terminus and the C-terminus and can be used to identify the weakest regions of the proteins. At these regions, there are low density of bonds. As the protein unfolds the *Q* value, the fraction of native contacts still intact, decreases and the protein loses rigidity. The shear which initially peaks at the few flexible residues, gradually becomes delocalized over the entire protein. In the native state (*Q* = 1 in Fig 1-B), the shear peaks at N-terminus and $\hbox {Loop}_1$ with N-terminus becoming floppy. When 30 bonds are broken we find the shear at N-terminus, $\beta _2$ and $\beta _3$ increases (*Q* = 0.82 in Fig 1-B) suggesting breaking of the bonds between N-terminus-$\beta _3$ and $\beta _2$-$\beta _3$. Later (*Q* = 0.7 in Fig 1-B) both $\beta _1$ and $\beta _2$ become floppy suggesting their loss of contact. All throughout the bonds of $\alpha$-helix remain stable. Fig 1-C shows that the thermal unfolding initiates at these weakest region of the protein like nucleation, then gradually it spreads to regions next to them and finally the shear is delocalized over most of the protein signaling that the entire protein becomes floppy. We explore if the shear in the native structure (*Q* = 1) can be used to predict the bond breaking sequence during unfolding. For each residue, we record the order in which the bonds of the residue with its neighbors are broken during unfolding. For each residue we take the average of the bond breaking order and find that there is a strong negative Pearson correlation of -0.79 between the logarithm of the shear in the native structure and the average bond breaking order in Fig 1-D, implying that the regions of high shear are more prone to bond breaking and that shear can be used to predict the bond breaking sequence or high shear regions have low average bond breaking order. The unfolding process involves the disruption of bonds, leading to the reduction in the average coordination number $<z>$ per residue and the increase in floppy modes for thermal unfolding shown in red in Fig 2-A. Force-induced unfolding is shown in blue and is detailed in the next paragraph. For both cases, in instances of high $<z>$, there are flat regions where alterations in $<z>$ do not affect the number of floppy modes. Initially, the protein is overly constrained due to an abundance of redundant bonds, and breaking these bonds does not generate new floppy modes. At later stages of unfolding $<z>$ less than 3, not many redundant bonds are left which lead to rapid increase of floppy modes. The changes in the number of floppy modes are gradual as compared to the sudden jump during the rigidity transition as observed in Ref^24^.

Alternatively, we unfold CI2 by pulling at their ends along x-axis direction in opposite directions as detailed in Sec 2.1. A question naturally arises whether a pulling force can trigger similar unfolding pathways as thermal unfolding. Pulling the residues at the N-terminus and C-terminus of 2CI2 causes uneven tension in the protein resulting in the bonds near both the termini becoming floppy (*Q* = 0.82 in Fig 2-B). Gradually the bonds between the N-terminus region with the $\beta _3$ and the bonds between the $\alpha$-helix and $\hbox {Loop}_1$ get ruptured (*Q* = 0.35 in Fig 2-B). The bonds within $\alpha$-helix and $\beta _1$ with its neighbor break making them flexible. The initial unfolding pathway is similar to the pathway observed during thermal unfolding. However, we find from the comparison of the shear in Fig 1-B and Fig 2-B that in thermal unfolding the protein becomes floppy from the hinge and in the force-induced unfolding from the N-terminus. This is a consequence of the fact that the $\alpha$-helix near the N-terminus unfolds first due to the proximity of the point of application of the force leading to unfolding predominantly from the N-terminus side. Our shear analysis shows that the unfolding pathways are different. These differences also show up in the behavior of the number of floppy modes with the coordination number in Fig 2-A. For force-induced unfolding also, we find a high correlation of 0.68 between log shear and the average bond breaking order (not shown). But the correlation is lower than that of the thermal unfolding.

**Fig. 3:**
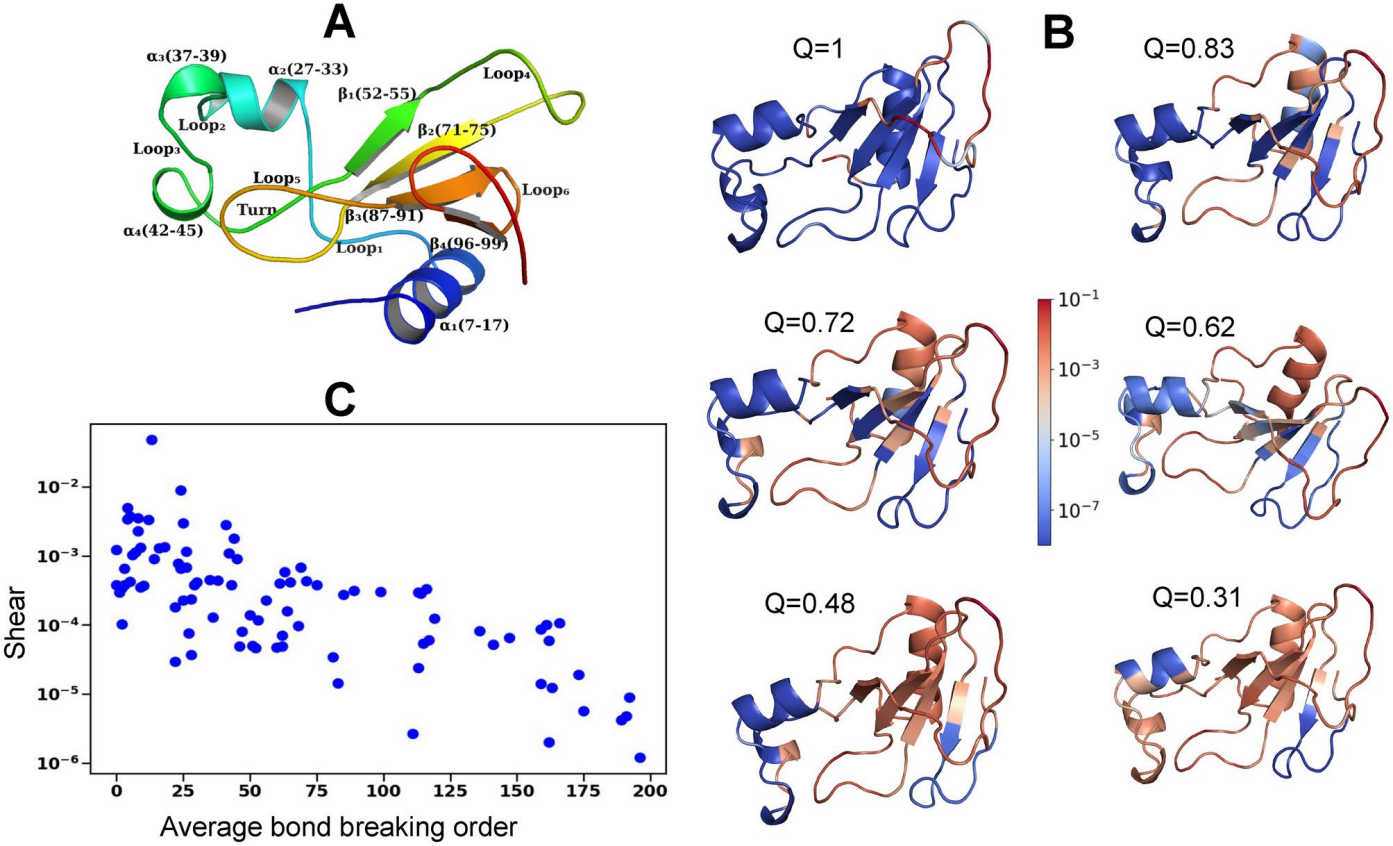
Thermal unfolding of Barnase. (**A**) The native structure of Barnase (PDB ID : 1a2p) showing the various secondary structures. (**B**) The shear at each residue during unfolding with regions of high (low) shear are shown in red (blue). (**C**) The shear for the residues in the native structure (*Q* = 1) vs the average order in which the bonds break for Barnase in a total of 200 bond breaking.

We study the thermal unfolding and the corresponding change in shear for another protein, Barnase. Barnase (PDB code 1a2p) is a ribonuclease with four $\alpha$-helices and a four-stranded $\beta$-sheet (Fig 3-A) forming three hydrophobic cores. Fig 3-B shows that in native state $\hbox {Loop}_4$ (*Q* = 1) and N-terminus have high shear and are floppy. Next $\hbox {Loop}_1$, $\hbox {Loop}_4$, $\hbox {Loop}_5$ and $\hbox {Loop}_6$ all become flexible at *Q* = 0.83 in Fig 3-B and further at *Q* = 0.72 and *Q* = 0.62, $\alpha _1$-helix opens up. At *Q* = 0.48 and *Q* = 0.31 the remaining helices, $\beta _1$, $\beta _2$ and $\beta _3$ and at the end $\beta _4$ become flexible resulting in the unfolding. We again find a strong correlation of 0.72 between the logarithm of the shear of the residues in the native state of Barnase and the average order at which the bonds with their neighbors are broken in Fig 3-C. Our results suggest that shear can be used to predict the bond breaking sequence for thermal unfolding but less so for the force-induced unfolding.

### Results on CI2 thermal unfolding

**Fig. 4:**
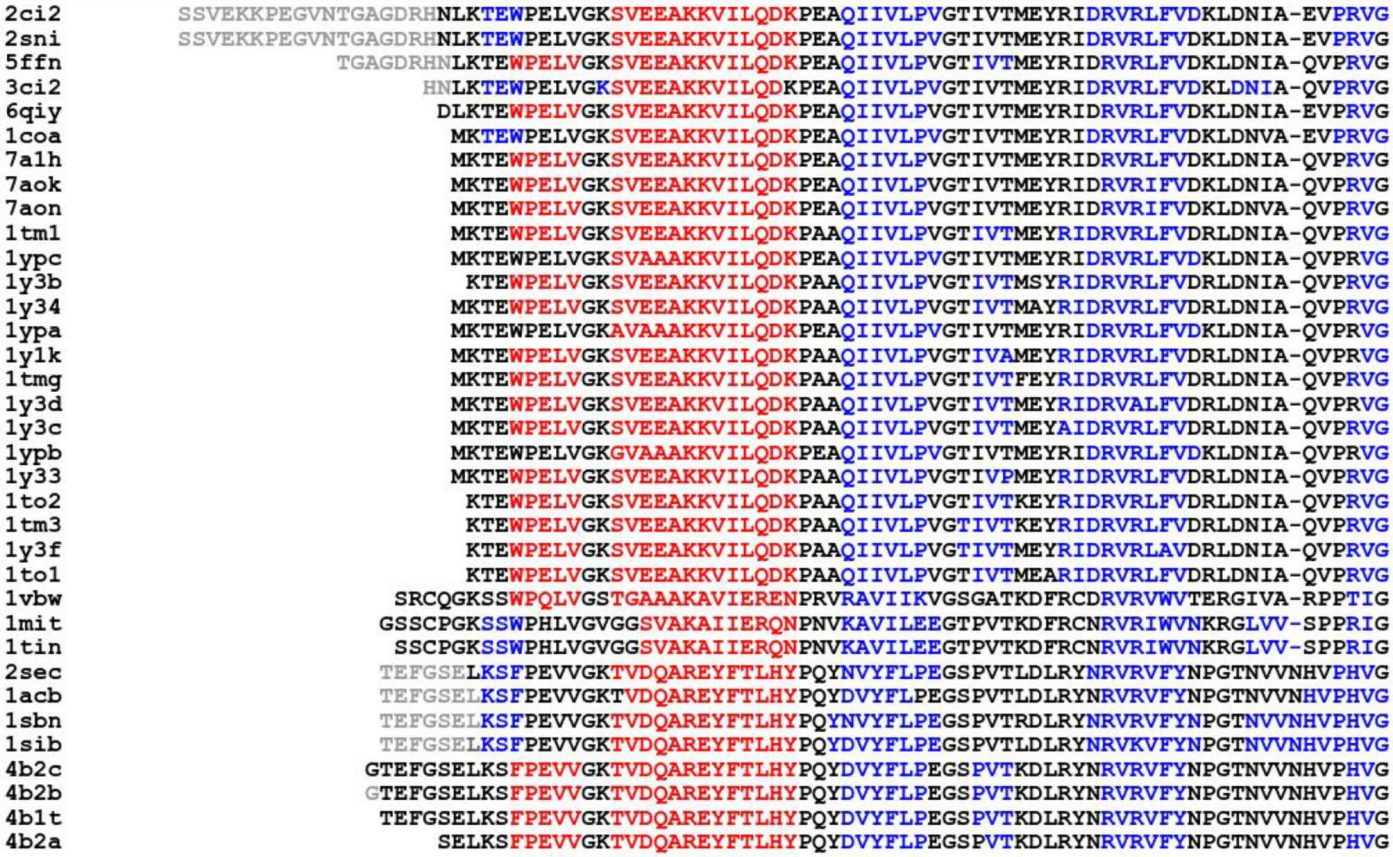
CI2 family sequence. The sequence alignment and the color coding of the secondary structures of the 36 proteins of the CI2 family (PDB IDs labeled on the left) aligned using protein BLAST. The color red denotes $\alpha$-helix, blue denotes $\beta$-strands and black denotes loops. The gray regions are regions lacking any secondary structures (disordered regions).

**Fig. 5:**
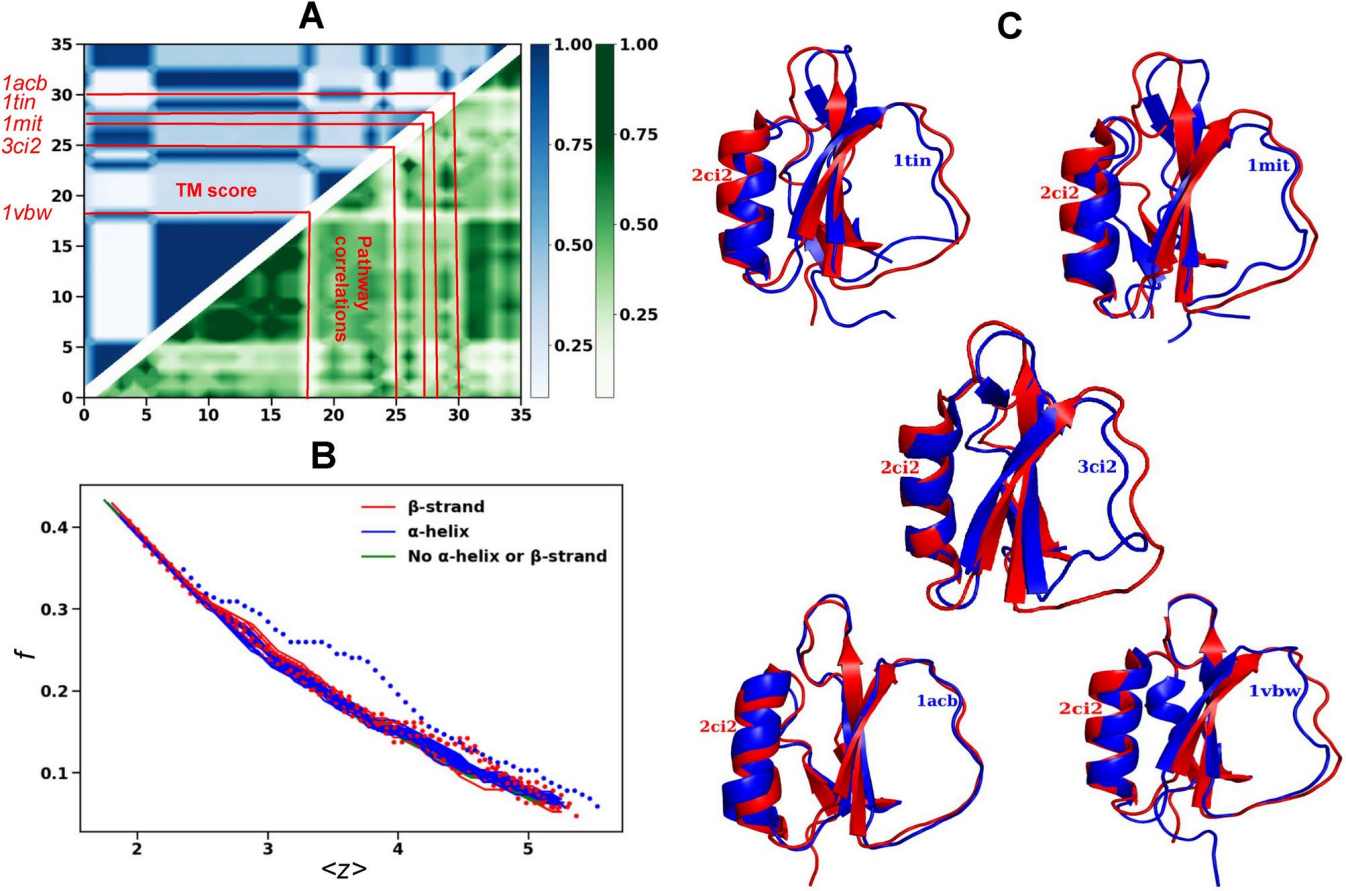
Thermal unfolding pathways for CI2 family. (**A**) TM-score measuring the structural similarity between two proteins is obtained from the optimized residue-to-residue alignment^63^. TM-score between each pair of 36 proteins of CI2 family is plotted in blue. Also shown in green are the correlations of the bond breaking sequences between each pair of member proteins. The trivial diagonal elements (TM-score / correlations of the unfolding pathways of a protein with itself) are not plotted. The first 23 proteins have a $\alpha$-helix at the N-terminus, next 10 proteins have $\beta$-strand at the N-terminus and the last 3 proteins have neither. (B) The behavior of the fraction of floppy modes *f* as a function of the coordination number $\langle z\rangle$ for the family members for the three types of proteins. The floppy curves exhibiting the greatest degree of variation are represented by dotted lines. We have marked these proteins by red lines in (**A**). (**C**) The native structures of the proteins with distinct floppy mode curves (dotted) in (**B**).

We simulate the unfolding pathways of 36 proteins of CI2 protein family with available PDB structures obtained from the PFAM database. Based on a coarse grained correlation ( Section 2.4) we compare the pathways for the member proteins and quantify the relationship between their native structures, the unfolding pathways and the number of soft modes. Fig 4 shows the sequence alignment of the members of the CI2 family with coloring based on the secondary structure. There are three types of proteins with similar secondary structures except with a 3-10 helix, a $\beta$-strand or lacking both at the N-terminus. Helices and $\beta$-strands that are part of the hydrophobic core are mostly conserved. We apply the thermal unfolding algorithm outlined in Section 2.1 and we use a vector representation of the bond breaking sequence (more details in Sec 2.4) corresponding to the five clusters of bonds in the contact map of CI2 in Fig 1-A. The covariance matrix, whose (*i*, *j*) th element quantifies the correlations of the bond breaking sequence between protein *i* and *j*, is calculated using the method in Sec 2.4 and is plotted in Fig 5-A. This is superimposed with a measure of structural similarity between two proteins, the TM-score described in Ref^63^ in Fig 5-A. TM-score is a measure of topological similarity of two proteins. It takes values between 0 and 1, with 1 indicating that the two structures perfectly match. The contour plot of the TM-score and the correlations between the unfolding pathways exhibit a qualitative resemblance. Five proteins 1tin, 1mit, 3ci2, 1acb and 1vbw, with divergent floppy curves, not only have low TM-score but also have low correlations between the unfolding pathways implying that the proteins with different native structures will have different unfolding pathways. The well-established principle that the native structure of a protein governs its folding pathway^64,65^ is confirmed by this finding. In Fig 5-B we find that the behavior of the number of floppy modes is most divergent initially when the coordination number is high, however after the coordination number drops below 3.7 their pathways become more convergent. The number of floppy modes depends on the entire protein and hence it is a global measure of the structural feature of the protein. The inflection point of the number of floppy modes has been used by Rader et. al.^24^ to obtain a sharp first-order-like rigid to floppy transition. However, in our case Fig 5-B shows more of a gradual behavior for these curves. The proteins with most diverging floppy curves in Fig 5-B are same proteins in Fig 5-A with low TM-scores and low pathway correlations: 1tin, 1mit, 3ci2, 1acb and 1vbw. The proteins with more coinciding or similar (dissimilar) floppy modes curves in Fig 5-B, have more similar (dissimilar) unfolding pathways implying that the floppy mode curves can be used to characterize the order of bond breaking between the secondary structures of proteins that leads to their unfolding. All these results imply that the floppy curves can serve as a tool to differentiate the unfolding pathways of proteins particularly the ones that are structurally different.

**Fig. 6:**
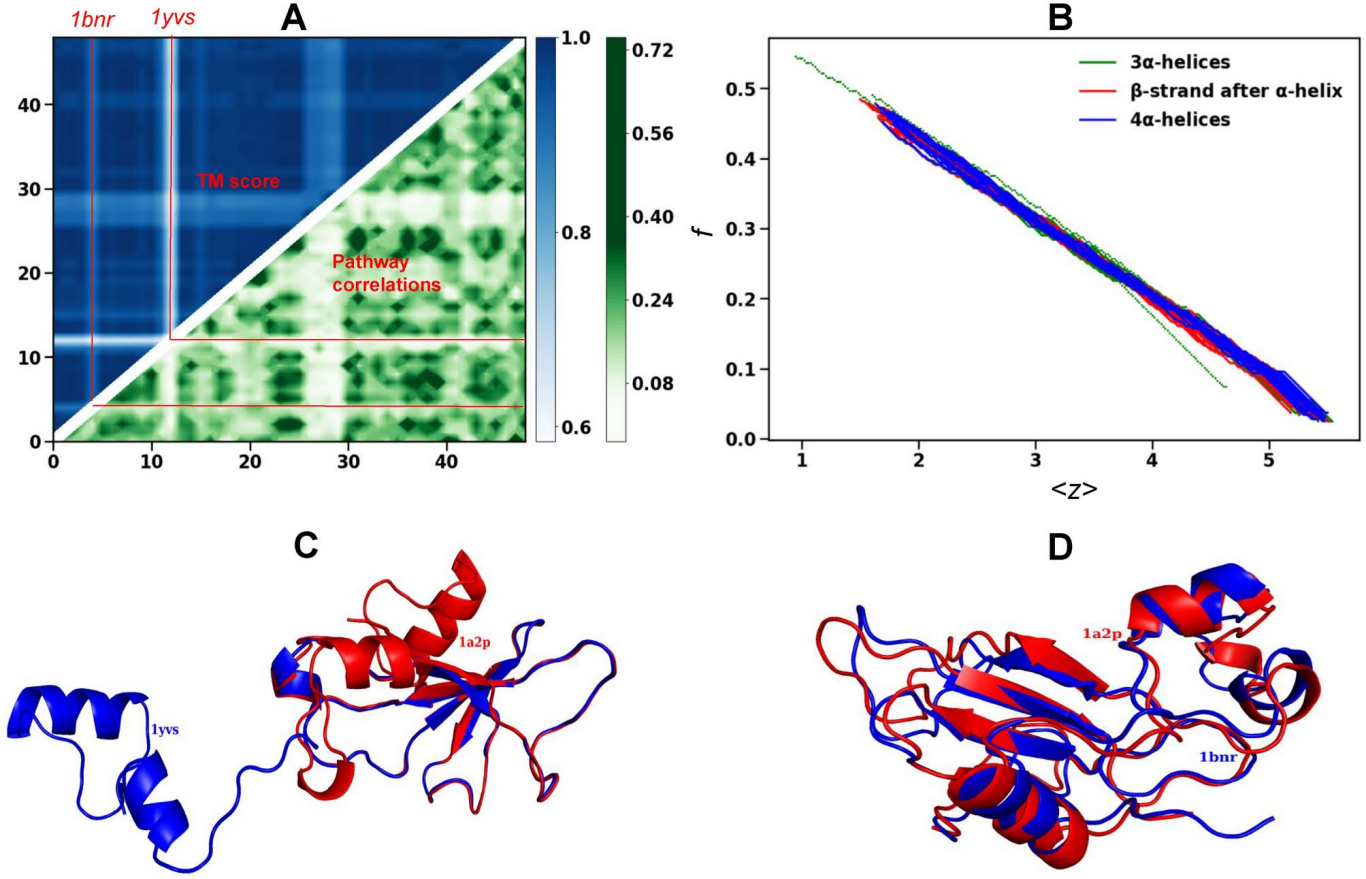
Thermal unfolding pathways for Barnase family. (**A**) TM-score between 49 proteins of Barnase family superimposed with the correlation between the unfolding pathways. The first 14 proteins have three $\alpha$-helices, next 19 proteins have $\beta$-strand after $\alpha$-helix and the last 16 proteins have four $\alpha$-helices. 1yvs and 1bnr have least correlated pathways with low TM-scores. (**B**) The floppy modes *f* as a function of $\langle z\rangle$ show largest variation for 1yvs and 1bnr (green dotted). (**D**) The structures of 1yvs and 1bnr superimposed with Barnase (1a2p).

We examine the structural characteristics of proteins to identify the factors leading to the most distinct pathways with minimal correlations in Fig 5-C. The superposition of the two proteins, 2ci2 and 3ci2 shows the structural differences between the two. 3ci2 has an extra $\beta$-strand in $\hbox {Loop}_2$ between $\beta _2$ and $\beta _3$ that causes strong interactions between $\alpha$-helix and $\hbox {Loop}_2$, and $\beta _2$-$\beta _3$ resulting in the bonds to break later. 1vbw has smaller $\beta _2$ and $\beta _3$ and has an $\alpha$-helix at the N-terminus unlike 2ci2. The bonds of $\alpha$-helix and $\beta _1$-$\beta _2$ break first which is the reverse of 2ci2. 1acb has larger $\beta _3$ and the N terminus-$\beta _3$ and $\alpha$-$\hbox {Loop}_2$ breaks earlier then $\beta _2$-$\beta _3$ unlike 2ci2. 1mit and 1tin has a much longer N-terminus than 2ci2 and the loop at this terminus has a very different orientation compared to the other proteins. The bond breaking order is similar to 3ci2 except that all the bonds in $\beta _2$-$\beta _3$ breaks in 1mit before $\beta _1$-$\beta _2$.

### Results on Barnase thermal unfolding

In Fig 6-A we illustrate the TM-score vs the correlations between the thermal unfolding pathways of the 49 members of the Barnase family. Both the contour plots exhibit similar qualitative features. Proteins 1yvs and 1bnr ( red lines) and proteins 2kf3, 2kf4, 2kf5 and 2kf6 (lighter band near 30) have significant differences in unfolding pathways as well as lower TM-score. The divergent behavior of these proteins, particularly for 1yvs and 1bnr (green dotted), also show up in the floppy curves in Fig 6-B. The native structures of these two proteins differ significantly from the other members (see Fig 6-C and Fig 6-D). They have 3 $\alpha$-helices making the hydrophobic core 2 weaker. Additionally we find that the hydrophobic core 1 is partially open in 1yvs with the $\alpha _1$ and $\alpha _2$ far from the rest of protein.

### Results on CI2 force-induced unfolding

**Fig. 7:**
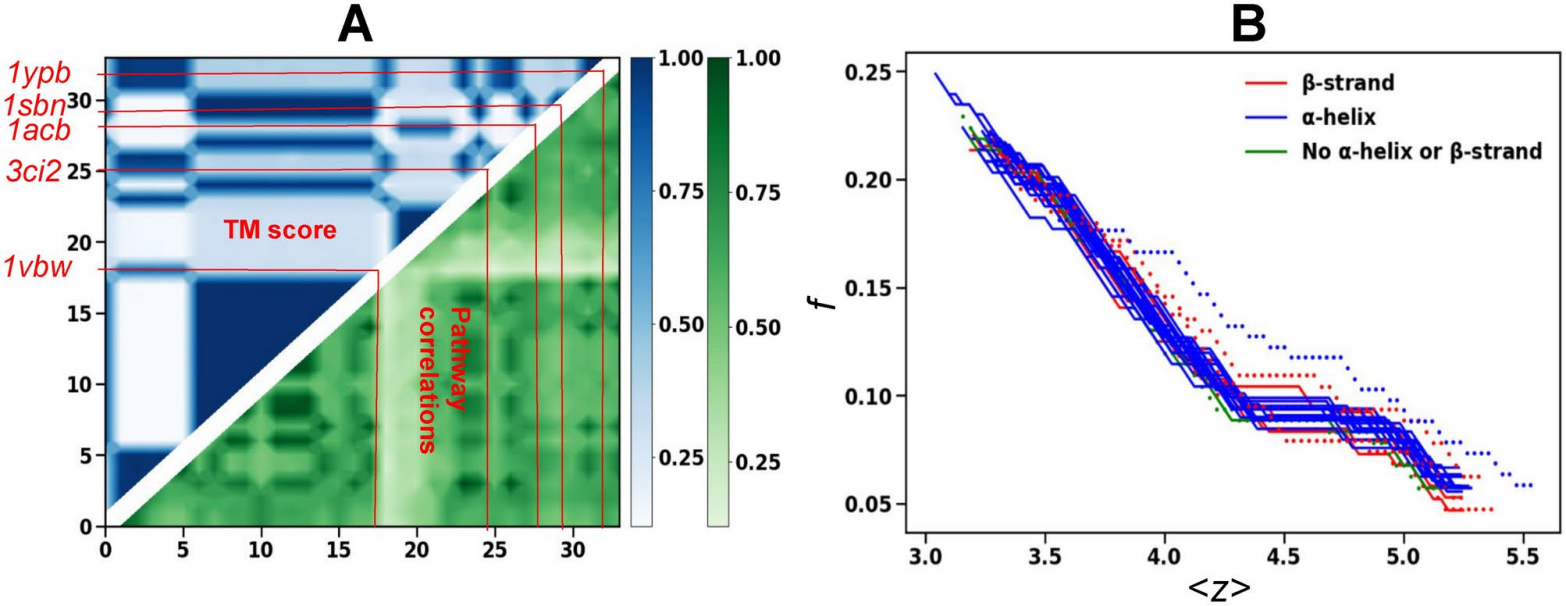
Force induced pathways for CI2. The TM-score between pair of proteins in CI2 family superimposed with the correlation between the unfolding pathways. The order of proteins are same as Fig 1-A. The most divergent floppy mode curves as a function of $\langle z\rangle$ are represented by dotted lines in (**B**) and by red lines in (**A**).

For force-induced unfolding of CI2 family proteins by pulling at the ends, we observe a minimal resemblance between the TM-score and the correlation of unfolding pathways in Fig 7-A. In fact the plot shows high correlations between the unfolding pathways for almost all the proteins. However, the floppy mode curves in Fig 7-B show significant differences for proteins 1ypb, 1sbn, 1acb, 3ci2 and 1vbw. It is important to note that most of these proteins show different unfolding pathways even for thermal unfolding.

## Conclusion

We investigated the changes in the local and global structure of small single domain proteins during thermal and force-induced unfolding. Coarse-grained simulations based on the Gaussian Network Model^43^ were used to obtain the unfolding pathways. The local structural features were captured using the shear part of the strain which peaks at the flexible regions of the protein. The high shear regions occur at the ends and at the hinge site of the proteins. We found strong correlations between the high shear and the order of bond breaking which suggests that the bond breaking starts at the high shear regions and spreads to regions of low shear until the entire protein becomes floppy.

We simulated the unfolding pathways for the members of CI2 and Barnase families. For thermal unfolding of both CI2 and families, the unfolding pathways have qualitative agreement with the TM-score implying that the structural dissimilarity lead to very different unfolding pathways. However for force-induced unfolding for CI2 this agreement is very weak. The force-induced unfolding pathways are very similar for almost all the proteins in the family even for low TM-scores.

Additionally, we explored how the global structural parameter, the number of floppy modes, behaves during unfolding. The number of floppy modes increases with unfolding and we found that the proteins which have similar unfolding pathways have converging behavior of these floppy mode curves, while the proteins with very different unfolding pathways have divergent floppy mode curves. This implies that the number of floppy modes serves as a tool to characterize variations in protein unfolding pathways. Analysis of the proteins’ 3D structures reveals that divergent floppy mode behavior stems more from the differences in their tertiary structures than their secondary structures.

Overall we find two tools: 1. the shear that contains the local structural information about the protein and 2. the number of floppy modes that contains the global structural information about the protein. The interplay of these two will be useful to study the structural transitions in biomolecules.

## Data Availability

All data generated or analyzed during this study are included in this published article.
